# Medical Management of Problematic Sexual Arousal for People With a Sexual Conviction in England and Wales: Challenges, Learning and Progress

**DOI:** 10.1002/cbm.70030

**Published:** 2026-03-21

**Authors:** Belinda Winder, D. Grubin, M. Underwood, Z. Antoniadis, M. Carvalheiro, E. Marshall, C. Norman, R. Bourne, A. Kaul

**Affiliations:** ^1^ Nottingham Trent University Nottingham UK; ^2^ Newcastle University Newcastle upon Tyne UK; ^3^ Devon Partnership NHS Trust Exeter UK; ^4^ Nottinghamshire Healthcare NHS Foundation Trust Nottingham UK

**Keywords:** evaluation, medical management, MMPSA, problematic sexual arousal, sexual conviction

## Abstract

**Background:**

In England and Wales, the primary treatments for individuals convicted of sexual offences are psychological. However, medication to manage problematic sexual arousal (MMPSA) is gaining importance as an alternative. This article reviews the current evidence surrounding the MMPSA approach.

**Aim:**

This paper synthesises challenges encountered, advancements achieved and learnings accumulated over 16 years of the MMPSA treatment pathway from 2009 to 2025 in England and Wales.

**Methods:**

Drawing on a programme of mixed‐methods research, including cohort studies, case studies, qualitative interviews with patients and professionals and implementation evaluations, this paper seeks to bring together key findings to present a consolidated picture of the research on the MMPSA pathway to date. The focus is on synthesising findings and identifying implications for service delivery.

**Results:**

Evaluations of treatment outcomes showed promising results regarding the effectiveness of the MMPSA service. Qualitative analyses and case studies provided insightful details regarding patient and staff concerns that may hinder the efficiency and reach of the treatment pathway. Research with community clinicians highlighted issues regarding the ‘off‐label’ use of medication for this purpose.

**Conclusions:**

The MMPSA treatment service is available in a limited number of prisons in England and Wales. Supported by promising service evaluations and existing literature, a larger population could benefit from MMPSA treatment. Furthermore, the MMPSA service would benefit from improvements to create smoother transitions for individuals leaving prison and entering the community, and it should ideally be expanded to ensure that those in the community can also access the MMPSA service.

## 1 Introduction

Problematic sexual arousal refers to a range of issues that include intrusive, persistent and intense sexual thoughts, persistent physiological arousal, unhealthy sexual urges and sexual behaviours that create distress and are difficult to manage. This paper uses the term as an umbrella concept to encapsulate these challenging experiences. We include paraphilic elements, such as paedophilic disorder (American Psychiatric Association [APA] 2013; World Health Organisation [WHO] 2019) or sexual sadism disorder (APA 2013; WHO 2019).

Elements of problematic sexual arousal have consistently been recognised as an important factor in theories and models of sexual offending behaviour (Finkelhor and Hotaling 1984; Seto 2019; Ward and Beech 2006; Ward and Siegert 2002); features such as sexual preoccupation, paraphilias, hypersexuality and sexual self‐management also serve as strong predictors of recidivism (Gregório Hertz et al. 2022; Hanson and Morton‐Bourgon 2004; Hanson et al. 2007; Mann et al. 2010; Seto et al. 2023, 2024).

## 2 The Medication to Manage Problematic Sexual Arousal (MMPSA) Service in England and Wales

The MMPSA service is a healthcare pathway that provides pharmacological treatments to adult men in prison who experience problematic sexual arousal (as defined above). It aims to help individuals manage intrusive sexual thoughts, hypersexuality and sexual compulsivity, seeking to improve wellbeing and enable better engagement in rehabilitation programmes. It is currently managed and delivered by the offender personality disorder pathway, a jointly funded partnership between HM Prison and Probation Service (HMPPS) and NHS England. The service offers voluntary patient‐centred support grounded in clinical assessment and ongoing monitoring.

The MMPSA service at HMP Whatton was instigated on the recommendation of a consultant psychiatrist (Kaul, co‐author) to the Governor of HMP Whatton in 2009. Kaul suggested that a specialist pharmacological treatment pathway be implemented to address problematic sexual arousal among people in prison. An initial service model was subsequently introduced at HMP Whatton, with the lead author tasked with evaluating its implementation and impact.

Following encouraging findings from an early outcome evaluation (Winder, Lievesley, Elliott, et al. 2014), which analysed data collected between 2010 and 2012, a formalised MMPSA service was established at HMP Whatton in 2014. In 2015, as part of the developing research programme, a patient and public involvement (PPI) group was established at HMP Whatton to ensure that ongoing research and service development were informed by lived experience. All members of the PPI group had a sexual conviction, and several had received MMPSA treatment.

Over subsequent years, the service expanded to several additional prisons across England and Wales. The associated research programme comprised multiple strands. Quantitative evaluations included service outcome studies (Winder, Lievesley, Elliott, et al. 2014; Winder et al. 2018), work examining problematic sexual arousal in the context of personality disorder (Hamilton 2020; Winder et al. in preparation), analyses of general practitioner (GP) prescribing practices (Marshall et al. in preparation) and research focusing on specific populations, including men with intellectual disabilities (Bradbury and Lievesley 2020). Qualitative research studies explored the experiences of patients (R. Lievesley et al. 2014; R. A. Lievesley 2019) and psychologists, prison and probation staff (R. Lievesley et al. 2024). In addition, broader service impact and implementation were examined through case studies (Winder, Lievesley, Kaul, et al. 2014) and the evaluation of wider system effects (Winder and Blagden 2020). Most recently, the evaluation programme incorporated a nonmedicated comparison group examined against the medications, which were further categorised into anti‐androgens (AAs) and selective serotonin reuptake inhibitors (SSRIs). This evaluation provided stronger evidence of both statistical and clinical reductions in problematic sexual arousal following pharmacological treatment (Winder et al. 2024).

In 2022, a pilot programme was launched in southwest England to develop a model of equitable provision across all prisons in that region. This pilot also introduced a community arm to the MMPSA service, aiming to ensure continuity of pharmacological treatment following release (Underwood and Winder 2025).

## 3 Protocol and Medications

MMPSA is a health intervention that uses medications such as SSRIs, AAs or gonadotropin‐releasing hormone agonists (GnRH agonists). Prescribing protocols have been developed to support clinicians in determining appropriate dosing, monitoring requirements and necessary investigations (Grubin 2018). These protocols are broadly consistent with international clinical guidelines for the pharmacological management of paraphilic disorders (Thibaut et al. 2020). SSRIs are indicated in cases where sexual rumination or feelings of sexual compulsion are prominent; in situations where hypersexuality is the presenting issue, AAs or GnRH agonists are recommended (Grubin 2018). However, GnRH agonists are prescribed in HMPPS settings in limited instances due to their relatively high cost, meaning that they are generally used only when a patient has completed a determinate custodial sentence and is approaching release. Initiating GnRH agonists at the point of release can be challenging due to difficulties ensuring continuity of treatment in the community after release. For patients who are hesitant to take AAs or GnRH agonists, SSRIs may be prescribed despite their less potent effect on reducing libido (Thibaut et al. 2020). Our research indicates that patients often find the relief provided by SSRIs encouraging, leading them to subsequently request AAs (Winder and Lievesley 2012). For many individuals, however, SSRIs provide relief from problematic sexual arousal.

Reducing the risk of harm to others is considered a beneficial secondary outcome, rather than the primary aim, of pharmacological treatment. UK prescribing guidelines emphasise that treatment decisions should prioritise patient wellbeing and foreground the individual's status as a patient within a healthcare framework, rather than primarily as an ‘offender’ (Grubin 2018). This position differs from some international protocols, which prioritise reduction of reoffending risk over health and wellbeing outcomes (Thibaut et al. 2020; Turner et al. 2022). Although MMPSA is primarily wellbeing focused, treatment decisions may legitimately consider risk‐related factors, particularly where individuals present with elevated scores on empirically established risk factors, provided that these are aligned with the patient's treatment goals and undertaken with fully informed consent.

## 4 Current Need

Understanding the prevalence of problematic sexual arousal is complicated by differences in terminology, diagnostic criteria, populations and methods used in studies, often leading to conflicting results. Some studies rely on self‐reports or estimates, whereas others provide more robust data. For example, Hamilton (2020) measured levels of sexual compulsivity in men with sexual convictions at HMP Whatton, with 45% meeting the cut‐off for initial referral to the MMPSA service. Hamilton (2020) provides the highest estimate for all sexual offence subtypes (45%) using a validated measure, the sexual compulsivity scale (SCS; Kalichman et al. 1994).

To address some of the challenges in providing a prevalence estimate, we analysed data from the MMPSA study (see Winder et al. 2024), which involved 94 adult male participants. These individuals formed a consecutive induction cohort, comprising men entering a prison housing individuals with primarily sexual convictions during their first two to three months of custody. It is important to note that these participants were not referred for MMPSA treatment; rather, they served as a comparison group for men undergoing treatment within the MMPSA pathway. We categorised the participants from the comparison group who met the clinical cut‐off for the MMPSA treatment pathway in two ways: (1) a total SCS score of 15 or higher (the ten‐item scale results in scores between a minimum of 10 and a maximum of 40), or (2) scoring 3 or 4 on any single item of the SCS. Twenty‐seven participants (28.7%) satisfied criterion (1), and an additional six (6.4%) met criterion (2), resulting in 33 participants (35.1% of the comparison group) meeting the clinical cut‐off for referral to the MMPSA treatment pathway.

Further insight is provided by a 2021 needs analysis conducted in HMP Ashfield, incorporating the Offender Assessment System (OASys), a structured risk and needs assessment tool used by HMPPS, psychological reports, staff interviews and security data, which identified 18% of residents as being sexually preoccupied. Although only 10 had requested MMPSA at the time, projected uptake was expected to expand to 20 residents—about 5% of the population. These findings, based on different assessment methods and distinct prison populations, suggest that the proportion of men serving a custodial sentence for a sexual offence who meet clinical thresholds for MMPSA may range between 18% and 45%. A provisional working estimate of 25% appears both conservative and practical when planning services. This figure acknowledges unmet clinical need, expected uptake and service capacity. However, systematic, multi‐site ascertainment over the coming years would help to refine this estimate. With over 15,000 men currently serving custodial sentences for sexual offences in England and Wales (Ministry of Justice and HM Prison and Probation Service 2025), around 3750 individuals may benefit from MMPSA if the service were more widely available. Although not all eligible individuals will participate—for reasons including stigma, medical contraindications or personal choice—this estimate offers a realistic foundation for service development and expansion planning, particularly in anticipation of improved access in community settings.

A recent online community study (Winder et al. in preparation) using the newly developed Problematic Sexual Interests Questionnaire (PSIQ) and the SCS recruited 463 participants who met a screening threshold (SCS ≥ 11), ensuring that the sample comprised individuals with some degree of sexual compulsivity. Within this sample, over 75% scored above the MMPSA‐relevant service threshold of 15 on the SCS, and 35% exceeded the more stringent clinical threshold of 24. In terms of specific sexual interests, 42 participants (9.1%) reported experiencing sexual thoughts, arousal or masturbation involving children in the past 30 days, whereas 94 (20.3%) reported the same in relation to adolescents. In addition, 48 participants (10.8%) reported having previously tried to seek help for issues related to their sexual thoughts, urges or behaviours. Of those who sought support, less than half (37.5%) rated the support as helpful. Many participants also reported experiencing distress and interference in daily life due to their sexual thoughts and arousal. These findings highlight a significant unmet need for support among individuals in the community who experience problematic sexual arousal, suggesting that MMPSA could also have significant relevance beyond custodial settings.

## 5 Evaluating Impact

Evaluations of the MMPSA treatment pathway have shown that MMPSA can effectively reduce hypersexual behaviour, sexual preoccupation and obsessive sexual thoughts, helping men manage their emotions and improve focus (Winder, Lievesley, Elliott, et al. 2014; Winder et al. 2018, 2024). According to the most recent evaluation (Winder et al. 2024), self‐reported sexual compulsivity scores were significantly and positively correlated with five of the six clinical measures of problematic sexual arousal (see Table 2 in Winder et al. 2024 for further detail). The findings from this study further validate the SCS by demonstrating its convergence with clinical measures for problematic sexual arousal, which have been used in several other studies (e.g., Winder, Lievesley, Elliott, et al. 2014; Winder et al. 2018). Significant, positive correlations were identified between clinical measures, including ‘strength of sexual urges’ (*p* < 0.01), ‘ability to distract from sexual thoughts’ (*p* < 0.01), ‘time spent thinking about sex’ (*p* < 0.01) and ‘feeling horny’ (*p* < 0.01) with both pre‐ and post‐medication SCS scores.

Qualitative studies have supported the use of MMPSA, revealing that men using both SSRIs and AAs reported several positive outcomes, including personal development, improved daily functioning and a redefined sense of self, alongside a notable decrease in problematic sexual arousal (R. A. Lievesley 2019; R. Lievesley et al. 2014; Winder, Lievesley, Kaul, et al. 2014).

The 2024 evaluation (Winder et al. 2024) showed that MMPSA led to statistically and clinically significant reductions in sexual compulsivity among men in prison for a sexual offence. Over a 3‐month period, mean scores on the SCS dropped by 29.5% for men prescribed anti‐androgens and by 31.4% for those taking SSRIs, whereas no significant change was observed in the non‐medicated comparison group. This absence of change was evident among comparison participants who entered the study with elevated SCS scores. Clinically significant reductions (i.e., from above to below service cut‐off thresholds) were observed in approximately 38% of medicated participants. These findings provide promising evidence for the effectiveness of MMPSA, particularly SSRIs, in reducing problematic sexual arousal among men in prison settings, although the findings should be interpreted cautiously due to the lack of matched controls, modest sample size and nonrandomised design.

## 6 Medication and Psychological Interventions

A review of treatments found that medication is sometimes combined with psychotherapy and additional interventions (Schmucker and Lösel 2017). Importantly, however, ‘psychotherapy’ in this literature typically refers to a broad range of psychological inputs (e.g., individual therapy, counselling, relapse‐prevention or trauma‐informed support), rather than access to a single specialised offence‐focused group programme. The necessity for psychological therapy might partly stem from historical trends favouring psychological interventions for addressing sexual offending (HM Prison and Probation Service 2022; Levenson et al. 2024). Although it seems logical to address unproductive cognitive patterns, such as negative attitudes and grievance thinking, to enhance problem‐solving skills, the timing and sequencing of such interventions warrant careful consideration.

For some individuals, problematic sexual arousal may impede engagement in psychological work. The intensity of intrusive sexual thoughts can make concentration and emotional regulation difficult (Akerman 2008; Grubin 2018). Some report that exposure to graphic narratives during group therapy can provoke inappropriate arousal, leading to feelings of embarrassment and humiliation that hinder their participation. This situation can foster feelings of inadequacy and a sense of being ‘untreatable’, as reflected in the personal accounts of some individuals (Akerman et al. 2014; L. E. Marshall and Marshall 2006; Winder et al. 2024).

Currently, the only structured psychological programme in England and Wales aimed at addressing offence‐related sexual interests is the Healthy Sex Programme (HSP). Although HSP is tailored for individuals with persistent offence‐related sexual interests, such as those related to children, it has limitations in addressing those with high levels of sexual arousal and sexual preoccupation. The sole evaluation study presents mixed findings regarding HSP's effectiveness in reducing future risk of harm (Freel and Wakeling 2023). HSP was not designed to treat problematic sexual arousal, and there are presently no interventions expressly focused on this issue.

It is also crucial to recognise potential conflicts that may arise with treatments. HSP includes a module involving guided masturbation alongside aversive conditioning techniques using ammonia as the aversive stimulus. When this component is delivered at the same time as MMPSA, potential tensions between the two interventions need to be considered. For instance, in our experience, individuals with lower levels of adaptive functioning may find it confusing to be instructed by one group of staff to masturbate using ammonia, while simultaneously being prescribed medication that makes orgasm more difficult or unattainable. To avoid placing patients in a potentially harmful and bewildering situation, fostering open communication between those administering the MMPSA service in prison and the HSP treatment manager is essential.

Another important reason for promoting open communication between psychology and healthcare is the critical need for access to psychological therapy while a patient is undergoing treatment with medication, such as MMPSA. In some cases, problematic sexual arousal masks underlying trauma (R. Lievesley et al. 2014). When medication reduces problematic sexual arousal, this coping mechanism may diminish, potentially leaving previously unprocessed trauma more psychologically salient. In our clinical experience, this can sometimes be associated with increased emotional distress, including heightened vulnerability to self‐harm in some individuals, particularly those with lower adaptive functioning or intra‐familial offending histories. This highlights the need for integrated psychosocial support and further systematic research to develop appropriate structures that ensure patient wellbeing.

The challenge remains that pharmacological treatments, such as MMPSA, are often initiated only after psychological interventions. Winder et al. (2018) found that 85% of patients had completed psychological interventions before opting for pharmacological treatment. However, MMPSA can play a valuable role as a preparatory step prior to the commencement of psychological interventions. A research study by R. Lievesley et al. (2014) showed that patients reported improvements in concentration and reductions in sexual preoccupation when using MMPSA. This provided them with greater cognitive ‘headspace’ to engage more effectively in psychological therapy.

This finding underscores the importance of a combined and flexible approach to treatment in which medication and psychological support are integrated where clinically indicated (Lösel and Schmucker 2017). Although psychological interventions address certain aspects of sexual offending, not all people experiencing problematic sexual arousal will require formal psychological treatment. For others, long‐term pharmacological support may be essential for maintaining control over sexual urges. The key consideration is therefore the timing and nature of intervention, ensuring that psychological input—whether in the form of structured therapy, trauma‐informed support or a strong therapeutic alliance with a psychiatrist—is available where needed. This approach avoids assuming universal psychological pathology while recognising that, for some individuals, problematic sexual arousal may function as a coping mechanism for unresolved trauma.

The suggested ideal time for referral to the MMPSA service is early in an individual's sentence. Our PPI group recommended that individuals screened early in their sentence may be more affected by their actions and motivated to understand the reasons behind their situation and to change; later in their sentence, people's thoughts turn to release, and they may be less likely to engage with something that implies they have unaddressed needs.

## 7 Treatment Journeys

Of those prescribed medication in the MMPSA treatment pathway, approximately 68% were still in the service 6 months later. However, overall, people had quite different treatment journeys (Winder, Lievesley, Kaul, et al. 2014). Some men wanted to stop using MMPSA as soon as possible, whereas others—particularly those with a long history of offending or offences against children—preferred to continue the treatment long term. No cases followed the same treatment progression, and many factors affecting whether someone will continue the treatment without disruption or whether they will continue with it at all.

## 8 Voluntary Provision and Proposals for Mandation

The MMPSA service is voluntary, meaning that medication is prescribed only with the patient's consent, and they can pause or stop treatment at any time. This service cannot be mandated as a condition for release on parole or imposed by a court. However, it is concerning that some participants in the service mistakenly believe that it is a compulsory requirement for their licence (R. Lievesley et al. 2024). Discontinuing MMPSA can raise concerns as well. Parole boards, probation officers and police may worry that if individuals stop taking these medications, it could lead to a resurgence of their urges and potentially result in reoffending (R. Lievesley et al. 2014). Patients themselves may fear that they will not be able to regain their normal sexual desire. Emerging evidence of post‐SSRI sexual dysfunction indicates that this concern may be valid (Ben‐Sheetrit et al. 2023). For healthcare providers, the challenge lies in balancing effective treatment with the risks of withdrawal and long‐lasting side effects.

Proposals to make the service mandatory, via parole conditions or court orders, raise serious ethical, clinical and practical concerns. MMPSA should not be, and is not, simply the prescription of medication; it is a comprehensive service encompassing psychiatric assessment, therapeutic support and patient‐led decision‐making. Imposing medication risks shifts this person‐centred model into a coercive regime, potentially eroding engagement. Coercive frameworks may exacerbate grievance thinking, a cognitive style in which individuals feel unfairly treated or persecuted, often externalising blame and justifying harmful behaviour (Barnett 2011). This thinking style is not only psychologically corrosive but also overlaps with dynamic risk factors for sexual recidivism, such as hostility (Mann and Hollin 2007; Seto et al. 2023), especially when individuals believe that they are being punished rather than supported (Levenson et al. 2024).

This coercion could also damage the therapeutic alliance, which is one of the most robust predictors of treatment success across mental health and forensic domains (Flückiger et al. 2018). A sense of trust, collaboration and voluntary participation is critical to effective rehabilitation, particularly among individuals with sexual convictions, who may already struggle with shame, secrecy and mistrust (W. L. Marshall et al. 2009). Lower recidivism rates have been observed among individuals on probation who report greater trust in their supervising officers, reinforcing the value of a strong working alliance as part of the responsivity principle in effective interventions (Sturm et al. 2022). Mandatory MMPSA risks replacing therapeutic rapport with compliance monitoring, reducing treatment to a form of behavioural control rather than an opportunity for internal change.

Practical implementation challenges further complicate the picture. Adherence becomes more difficult to monitor without therapeutic buy‐in, particularly as SSRIs do not currently exist in depot form and rely on daily oral ingestion. The challenges of ensuring compliance, developing reliable and ethical monitoring systems and allocating sufficient resources for medication, specialist staff, training and comprehensive support services are considerable, particularly within the context of an already strained public sector.

The ethical objections to mandatory medical treatment are formidable, centring on the fundamental principles of informed consent, patient autonomy and bodily integrity (Appelbaum 2007). Forcing individuals to undergo medical interventions, particularly those with significant side effects, challenges the core tenets of medical ethics and human rights. It also places clinicians in a deeply conflicted role, potentially transforming them from therapeutic agents into instruments of state control.

Legally, a mandatory scheme would face significant hurdles. Its compatibility with existing UK legislation, particularly the Mental Health Act 1983 and c. 20 (1983) and the Mental Capacity Act 2005 and c. 9 (2005), is questionable if the targeted condition is not clearly defined as a qualifying mental disorder or if individuals retain decision‐making capacity. Furthermore, any such scheme would be rigorously tested against international human rights standards, notably Articles 3, 5, and 8 of the European Convention on Human Rights (Council of Europe 1950).

Additionally, determining when MMPSA would cease under a mandatory system is unclear. Would it be lifelong? Linked to psychological assessments? Parole decisions? This ambiguity could compound patient anxiety, especially given known long‐term risks such as post‐SSRI sexual dysfunction (Ben‐Sheetrit et al. 2023). A recent work by Wolba et al. (2025) highlights that discontinuing testosterone‐lowering medication in this population is complex, involving clinical, psychological and legal considerations. The development of the COSTLow‐R (Checklist for the Offence‐Specific Treatment – Low Risk version) reflects this complexity. Designed as a structured professional judgement tool, it supports clinicians in assessing readiness to reduce or discontinue pharmacological treatment by considering dynamic sexual risk factors, treatment engagement, offence‐related attitudes, psychological stability, and broader risk management factors. Importantly, it is not a mechanistic threshold tool, but a framework to guide multidisciplinary, individualised decision‐making. Its existence underscores that cessation decisions cannot be reduced to time served or biochemical markers alone, reinforcing the need for clear and proportionate discontinuation frameworks within any mandatory MMPSA system.

The debate is often framed as a dichotomy between voluntary and mandatory provision. However, existing sentencing frameworks in England and Wales suggest a potential third model. For example, drug rehabilitation requirements and mental health treatment requirements operate as court‐imposed conditions while still requiring patient consent and clinical oversight. Such hybrid approaches blur the distinction between coercion and voluntariness, raising important questions about how MMPSA might be structured within existing legal frameworks. Whether such a model would preserve therapeutic integrity or introduce similar ethical tensions remains an open question requiring careful consideration.

## 9 Community Treatment

For individuals who require ongoing treatment, it is essential that medication is easily accessible within the community. In several cases where individuals contacted the lead author after their release, they reported having stopped their medication due to the difficulties and embarrassment associated with requesting MMPSA in a community setting. Our research study indicates that men often struggle to access MMPSA in the community. They cite a lack of resources, a lack of confidence or willingness from GPs to prescribe medication, insufficient shared care agreements and feelings of embarrassment when asking their GPs for MMPSA medication. Additionally, many are hesitant to discuss their problematic sexual arousal with GPs (Marshall et al. in preparation; Winder 2016). Beyond access to medication itself, the availability of ongoing specialist clinical monitoring and support is critical to ensure appropriate use and to manage potential side effects.

In the UK, the initiation of MMPSA typically occurs in custodial settings by a psychiatrist. Individuals seeking help for problematic sexual arousal within the community typically start by consulting their GP (Hinchliff et al. 2018). However, SSRIs are not currently recognised by National Institute for Health and Care Excellence (NICE) guidelines for treating problematic sexual arousal (National Institute for Health and Care Excellence (NICE) 2015). Consequently, prescribing SSRIs in this context is considered ‘off‐label’, although it is permitted if necessary to meet the patient's needs (General Medical Council 2022). Marshall (in preparation) conducted qualitative research to explore the perspectives and experiences of community‐based GPs regarding prescribing MMPSA. The findings revealed a lack of experience, which in turn led to a corresponding lack of confidence among GPs in treating patients in primary care settings. Opinions on off‐label prescribing of SSRIs varied among the GP participants. Although some felt confident in prescribing the medication off‐label due to their familiarity with it, others were hesitant, particularly in such a sensitive area. Participants noted that having oversight from secondary care through a shared‐care agreement would enhance GPs' confidence in managing problematic sexual arousal in primary care. However, this presents a challenge, as most people requiring MMPSA do not have a mental disorder of a severity that would qualify them for secondary care mental health services. Moreover, these services typically lack the specific expertise needed to support the use of MMPSA in the community.

Patients accessing MMPSA in the community face several additional challenges. Many of these patients require consultations with specialists, and GPs often hesitate to prescribe medication for individuals with a sexual conviction or to see these patients in their clinics. This reluctance is compounded by the shame and stigma associated with sexual convictions, which can prevent individuals from seeking help. Furthermore, there is a significant lack of knowledge and understanding regarding people with sexual convictions. Additionally, as mentioned earlier, the primary type of medication used in the prison‐based MMPSA service is SSRIs, which are prescribed for the vast majority of patients. However, due to the difficulty of obtaining an SSRI prescription specifically for managing problematic sexual arousal, anecdotal feedback from members of our PPI group, along with participants in our studies, suggests that some men may attempt to be prescribed SSRIs for depression to benefit from the medication's antilibidinal effects.

## 10 Future Work

To date, there has only been one randomised controlled trial examining SSRIs and sexual compulsivity, with this study demonstrating significant treatment effects for reduction in sexual desire, frequency of masturbation and pornography use (Wainberg et al. 2006). However, we are now in the process of setting up FASAR (fluoxetine assisted sexual arousal reduction), a double‐blind randomised controlled trial, funded by the National Institute for Health and Care Research (ref: 151549), to evaluate the effectiveness of an SSRI (fluoxetine) in reducing problematic sexual arousal, compared to a placebo. The trial will take place across at least eight prison sites in England and Wales and data collection is due to commence in Spring 2026. Patients will be assessed for problematic sexual arousal, wellbeing, quality of life, and paedo/hebephilic interests, both before treatment and at three‐ and 6‐month follow‐ups. The primary outcome will be a measure of sexual compulsivity at the 6‐month follow‐up. Health‐economic data will also be collected.

## 11 Conclusion

The MMPSA treatment service is currently available in a limited number of prisons in England and Wales. This highlights a significant unmet need for MMPSA services beyond the existing prison system. To improve the MMPSA service, it could be beneficial to (i) ensure better integration of psychological and pharmacological treatments, (ii) facilitate smoother transitions for individuals leaving prison and (iii) expand provision to allow access for those in community settings. One of the main challenges of providing MMPSA in the community is the lack of a strong evidence base to support the on‐label prescribing of SSRIs in these settings. The FASAR trial may provide the necessary evidence to facilitate this change, but there is a need for education to encourage patients who could benefit from the service to come forward. Crucially, clear health‐based commissioning of MMPSA services—guided by a structured pathway approach—is needed to support effective and sustainable delivery in the community.

## Data Availability

The data that support the findings of this study are available on request from the corresponding author. The data are not publicly available due to privacy or ethical restrictions.
